# Malignant Peritoneal Mesothelioma: Clinicopathological Characteristics of Two Cases

**DOI:** 10.1155/2014/748469

**Published:** 2014-03-04

**Authors:** Mustafa Cem Algın, Faik Yaylak, Zülfü Bayhan, Figen Aslan, Nilüfer Araz Bayhan

**Affiliations:** ^1^Department of General Surgery, Faculty of Medicine, Dumlupinar University, 43100 Kutahya, Turkey; ^2^Department of Pathology, Dumlupinar University-Evliya Celebi Training and Research Hospital, 43059 Kutahya, Turkey; ^3^Department of Anesthesiology and Reanimation, Dumlupinar University-Evliya Celebi Training and Research Hospital, 43059 Kutahya, Turkey

## Abstract

*Introduction*. Peritoneal mesothelioma is a rare tumor, presenting difficulties in diagnosis and treatment. Peritoneum is the second most common area of the mesothelioma after pleura, and even synchronous pleural and peritoneal mesotheliomas are observed in 30–45% of all cases. The diagnosis may be difficult due to lack of specific symptoms and clinical findings. In addition, a delay in the diagnosis is not rare especially in the absence of previous asbestos exposure. Here we report two cases of malignant peritoneal mesotheliomas. The diagnostic and therapeutic approaches for these rare neoplasms are discussed. *Case Presentation*. The cases were two men (one aged 54 years old and the other 40 years old). Prolonged abdominal pain and swelling were the primary presentation symptoms and findings. The mesotheliomas were developed in the right upper quadrant of abdomen in both of the cases. Both cases were treated with surgical resection. Final diagnosis were possible with histological and immunohistochemical documentation of tumor characteristics, which were consistent with dictating a mesothelial origin. No history of asbestos exposure was reported. *Conclusion*. Peritoneal mesotheliomas are rare clinical entities. However, patients with prolonged abdominal pain and abdominal masses should be considered to have atypical pathologies such as peritoneal mesotheliomas.

## 1. Introduction

Malignant peritoneal mesothelioma is an uncommon disease and has a very poor prognosis with an average survival time between 5 and 12 months [1]. Inadequate and delayed treatment is the main reason for this short survival time. The incidence is about one in a million. Nearly one fifth to one third of all mesotheliomas is localized in the peritoneum. Fifty percent of reported cases have a history of asbestos exposure [1]. The delayed diagnosis of peritoneal mesothelioma is common due to a very long time interval between initial asbestos exposure and the onset of the symptoms. In addition, generally, the presenting symptoms are mild and nonspecific. Here, we report two extremely rare cases of epithelioid malignant mesothelioma and clinical presentation and outcomes have been discussed.

## 2. Case Presentation

**Figure 1:**
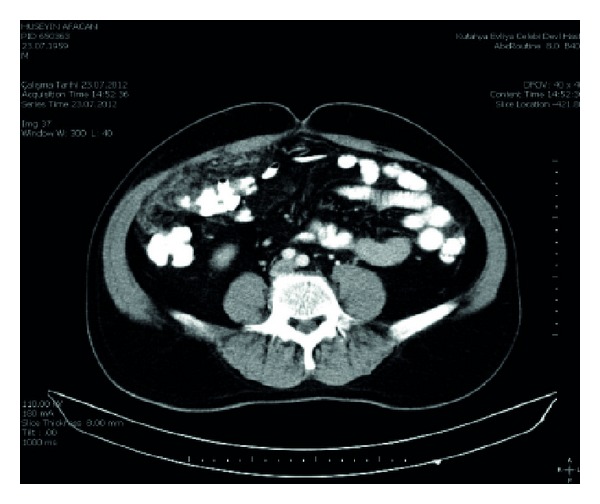
Omental infiltration and suspected invasion of hepatic flexure and right transvers colon.

**Figure 2:**
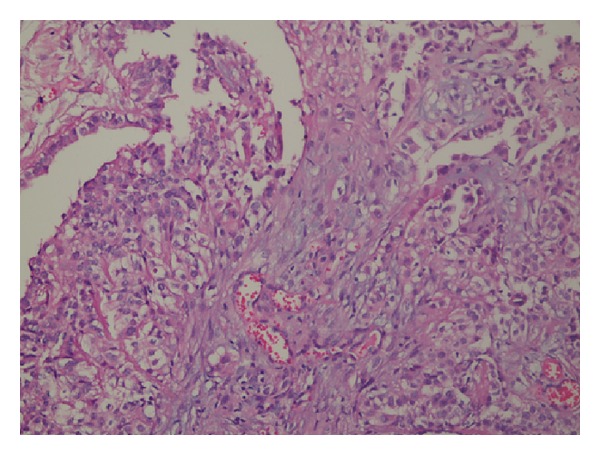
Malignant mesothelioma (H&Ex200).

Case 1: a 54-year-old male patient was admitted with complaints of shortness of breath simultaneous abdominal pain, stiffness, and swelling. The patient was referred to specialist pulmonologist. Thorax CT scan revealed the presence of common pleural effusion on the right side of the patient and changes in the lung fields adjacent to the effusion revealed the presence of passive atelectasis. The presence of collapse and consolidation was revealed in the right lateral segment lung. Thoracic surgery consultation was requested for the patient because of the right pleural effusion. Thoracentesis was performed to the patient by the thoracic surgeon. Cytological examination of the material was reported to be compatible with malignant epithelial tumor. However, distinction of mesothelioma and carcinomatous adenocarcinoma could not be made as a result of cytopathology. He had no known previous exposure to asbestos. In abdominal examination, an abdominal mass was palpated in the right upper quadrant. Abdominal CT was also performed to the patient because of prolonged abdominal pain. In abdominal CT imaging, suspected invasion of omentum has been found in hepatic flexure and right transvers colon (Figure 1). In addition, suspected infiltration and signs of compression of these colonic segments were determined. Laparotomy was scheduled for the intraabdominal mass. Mass of the omentum adherent to the hepatic flexure was found in the operation. After dissection of adhesions, the patient underwent total omentectomy. There was no other pathology in the abdomen. After recovery, the patient was discharged. Histopathology revealed malignant mesothelioma (Figure 2). Patient was referred to medical oncology after the period of recovery. Chemotherapy was administered to the patient by the medical oncology specialist. Control abdominal CT was performed after six cycles of chemotherapy. Two cystic lesions were detected in CT. One of them was in size of 18 × 12 cm and located inferior of the right lobe of the liver and the other one was in size of 10 × 8 cm and located in right inferior quadrant. Percutaneous drainage was performed for both cysts. The patient was hospitalized until the cysts completely collapsed. The patient was discharged after recovery. Patient is in follow-up period.

**Figure 3:**
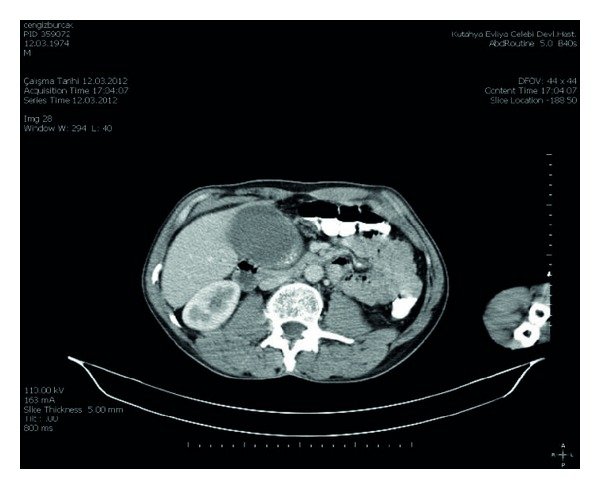
7 × 5 cm mass originating from the gallbladder.

**Figure 4:**
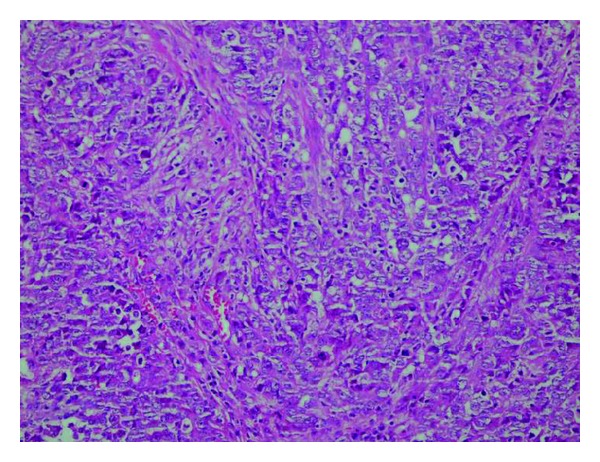
Epithelioid type of malignant mesothelioma formed by malignant epithelioid cells (H&Ex400).

In the second case, a 40-year-old male patient was admitted with abdominal pain that has continued for 2 months and abdominal swelling in the right upper quadrant. There was a palpable mass in the right hypochondrium in the physical examination. In the size of 7 × 5 cm, mass originating from the gallbladder was detected in the abdominal CT (Figure 3). The decision of the operation was given for the patient. The gallbladder was greater than normal size and in size of 7 × 5 cm, and tumoral mass was found originating from the inferior wall of the gallbladder. The patient had undergone cholecystectomy and resection of liver segment adjacent to gallbladder. The histopathology revealed epithelioid malignant mesothelioma (Figure 4). The patient was discharged and referred to medical oncology after the recovery.

## 3. Discussion

Mesothelioma is defined as neoplasm arising from the mesothelial cells which line the serous cavities such as pleura and peritoneum. Pleural mesothelioma is common and well known. However, isolated peritoneal mesothelioma is rare and presence of mesothelioma in atypical locations such as tunica vaginalis of the testis has been reported [2, 3]. The relation between asbestos exposure and the development of mesothelioma in pleura, peritoneum, and pericardium has been determined previously and cumulative asbestos exposure has been reported to be directly proportional to risk of cancer [1]. In addition, simian virus 40 has been named among etiological factors in carcinogenesis of mesothelioma [2, 4].

Patients with mesotheliomas do not present with distinctive symptoms and this causes difficulties in diagnosis and treatment. Two types of symptoms are generally reported in the patients with mesotheliomas: (1) abdominal pain, which is usually localized and related to a dominant tumor mass with little or no ascites and (2) ascites and abdominal distention without abdominal pain. Patients with mesotheliomas have been reported to generally present with one of these two types of symptoms and signs [5].

Imaging is important in diagnosis, and ultrasonography and CT scan of the abdomen are useful. However, pathological examination of biopsy or resection material is essential for confirmation of the definitive diagnosis. Open or laparoscopic surgery may be used to biopsy or with respective purposes for the suspicious lesions. Even laparoscopy may be very necessary in the diagnosis of some rare cases with ascites, such as primary mesothelioma. These clinical entities are usually overlooked by other diagnostic modalities, such as ultrasound and CT or ascitic fluid cytology. Laparoscopy may document the presence of the tumor fluid. However, there is controversies such as the potential that laparoscopy can facilitate tumor dissemination to port sites [6].

Prognosis is determined by the clinical presentation, the completeness of cytoreduction, and gender of the patient. It has been reported that women survive longer than men with this condition. Prognosis appears to be improved with the use of intraperitoneal chemotherapy. Over the past decade, the management of these patients has evolved similarly to ovarian cancer treatment. Currently, cytoreductive surgery, heated intraoperative intraperitoneal chemotherapy with cisplatin and doxorubicin, and early postoperative intraperitoneal paclitaxel are used. Adjuvant intraperitoneal paclitaxel and second-look cytoreduction are recommended after these perioperative treatments. This multimodality treatment approach with cytoreductive surgery and intraperitoneal chemotherapy has been reported to result in a median survival of 50 to 60 months [2, 5].

## 4. Conclusion

These cases indicate the importance of considering the rare clinical conditions such as peritoneal mesotheliomas in the patients with common clinical problems such as abdominal pain, mass, and ascites. Peritoneal mesothelioma is an uncommon disease, but should be considered in people presenting with abdominal mass, prolonged abdominal pain, and ascites, especially in those where the initial diagnosis is not clear. A history of asbestos exposure may not be present. Radiological investigations may overlook the diagnosis. Thus, a thorough clinical evaluation and a comprehensive approach are essential in order to diagnose and properly treat the potential patients with peritoneal mesotheliomas.
